# Supplementary material for: RNA Sequencing-Based Whole-Transcriptome Analysis of Friesian Cattle Fed with Grape Pomace-Supplemented Diet
Source: Animals (Basel). 2018 Oct 23;8(11):188. doi: 10.3390/ani8110188 (PMC6262483; doi:10.3390/ani8110188)
Supplement: Supplementary file 1 [file animals-08-00188-s001.pdf]

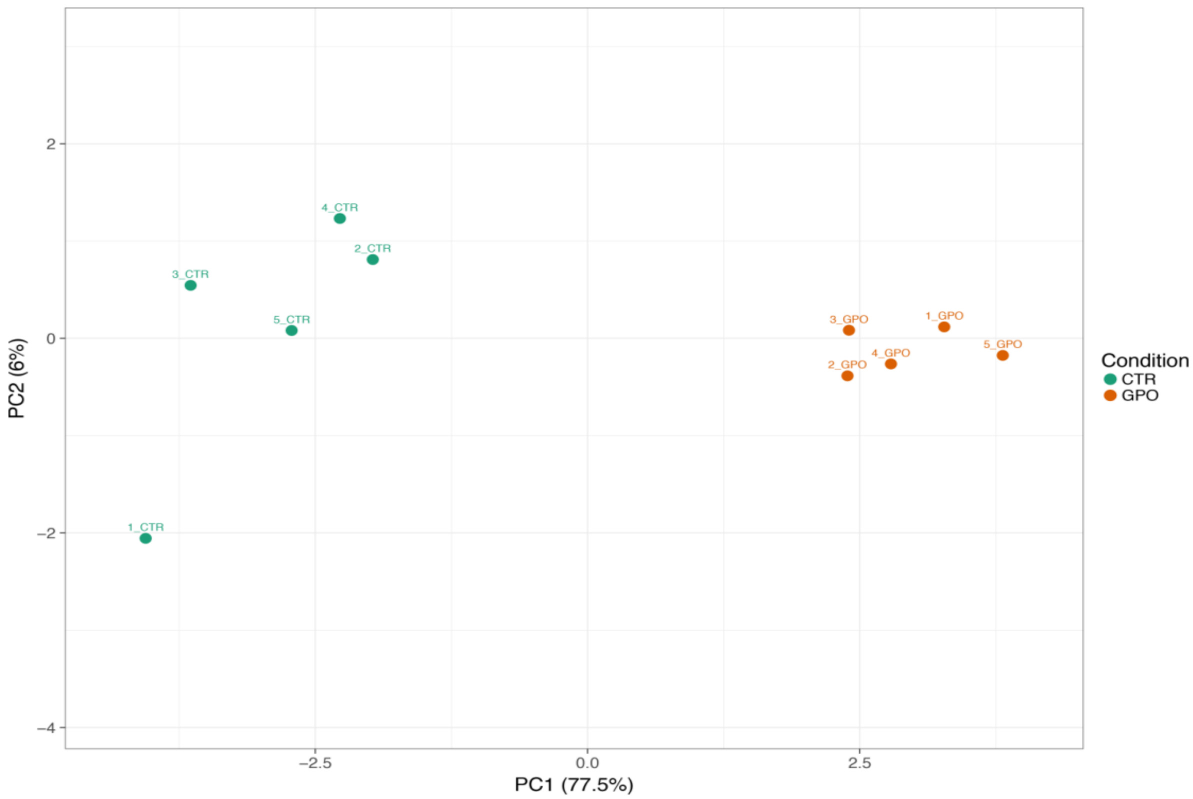

Figure S1. Principal component analysis (PCA) plot of the differentially expressed genes (DEGs) in veal calves after 75-days of grape pomace (GPO) supplementation, compared with unsupplemented control group (CTR). The GPO-supplemented animals (orange circles, right side) are separated from the unsupplemented CTR animals (green circles, left side) with the first two components (PC1 and PC2) accounting for 83.5% of the total variation. For interpretation of the references to color in this figure legend, the reader is referred to the web version of this article.

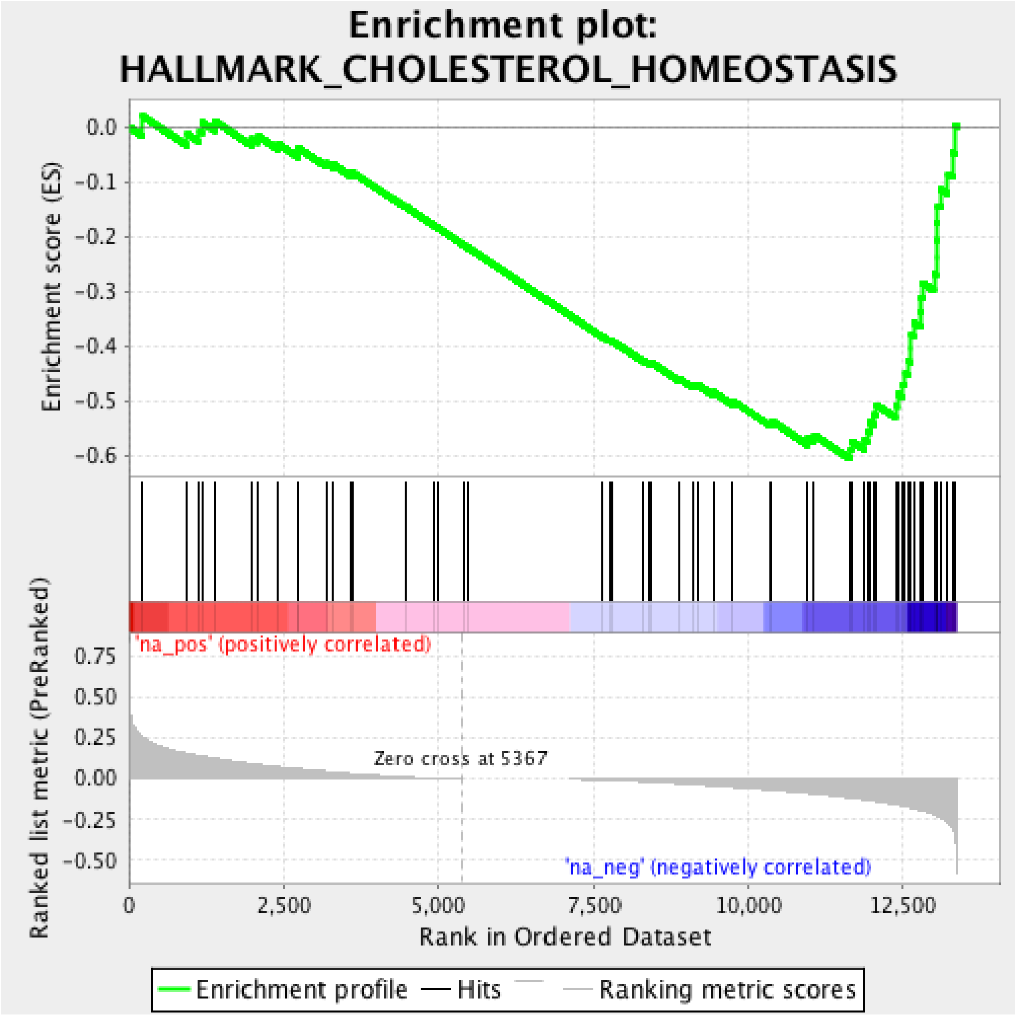

Figure S2. Gene Set Enrichment Analysis plot. GSEA was performed with either the canonical pathway or biological process gene sets in GSEA Molecular Signature Database.

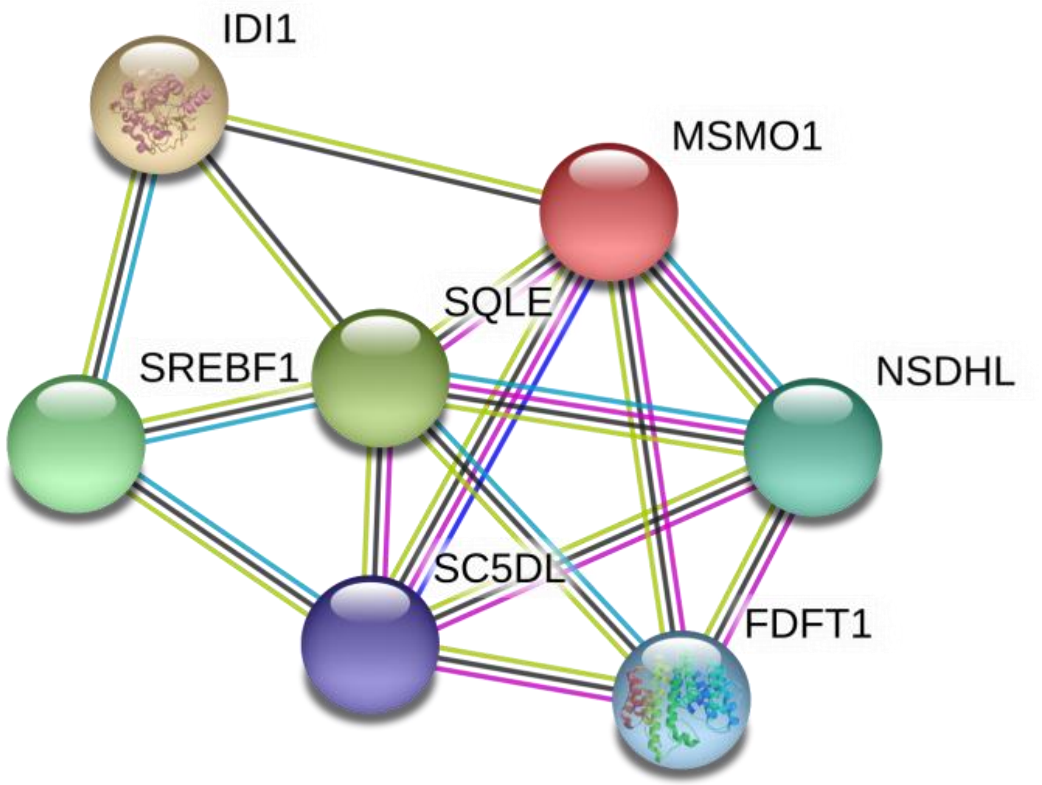

Figure S3. Network analysis (STRING software) of downregulated DEGs belonging to cholesterol biosynthesis pathways. The obtained interaction score (confidence limit) was 0,9 (the highest). Different line colors indicate the type of available evidence for the putative associations: green line, neighborhood evidence; purple line, experimental evidence; blue line, co-occurrence evidence; light-blue line, database evidence.

Table S1. Differentially expressed genes (DEGs) in Grape Pomace (GPO)-supplemented veal calves compared with unsupplemented animals.

Gene Symbol	Log2fc	Adj. *p*-Value
ABCB10	0.320834	0.008327122
ACAD10	0.250412	0.023255753
ADCYAP1R1	0.48731	0.000407042
ADD1	0.216388	0.005548905
ADORA2B	-0.25724	0.013231171
AGBL5	0.28767	0.016275577
ANKRD49	-0.29314	0.023108418
AOC3	-0.31824	0.023148283
AP3M1	-0.28135	0.003406127
APIP	0.417619	0.000142845
ARFIP1	-0.22331	0.009636664

ARHGEF10L	-0.29549	0.025678371
ARSH	0.230791	0.023191155
ATG3	-0.42196	0.003484141
ATP4B	0.32246	0.019837855
ATP8	0.348565	0.011165652
ATP8B2	0.233294	0.025968961
BBS7	0.278075	0.022406733
BCL10	-0.26131	0.001889871
BCL9L	0.280656	0.017985786
BCLAF3	-0.24987	0.01808778
BHLHE40	-0.29852	0.019702645
BOLA-DQB	-0.34693	0.001763759
BRCA1	0.289398	0.021092572
BREH1	0.348068	0.019576799
BSND	0.157955	0.025722594
C7H5orf15	-0.21973	0.020368992
CCDC114	0.3155	0.023671053
CCDC141	0.260829	0.01856064
CCDC8	-0.18	0.019060592
CCDC80	-0.33104	0.008207596
CCNL1	-0.29767	0.020980383
CCR10	0.429898	0.002815971
CD200R1L	-0.41996	0.000135854
CD276	0.482668	0.000656639
CD55	-0.32445	0.018339858
CD69	-0.25887	0.023544301
CD7	0.349588	0.015996526
CDAN1	0.267175	0.025313654
CDH20	0.305447	0.021584825
CDH24	0.335311	0.023125518
CEP85	0.223066	0.023742905
CHIC2	-0.26975	0.014335751
CHRNA9	0.222484	0.019341196
CISH	-0.59003	2.33E+06
CKAP5	0.198541	0.010866476
CLASP1	0.316006	0.001782444
CLCN6	0.326974	0.012088826
CLEC12A	-0.4291	0.003659709
CLIP2	0.30616	0.022392709
CMTM8	-0.4292	0.002995151
CNTNAP1	0.442979	0.003242019
COL18A1	0.375011	0.011957073
COMMD5	-0.21082	0.011532516

COPS4	-0.21878	0.015289512
CPA3	-0.40345	0.004870552
CPZ	0.33892	0.023579771
CUBN	0.376967	0.012214295
CYB5D1	0.291233	0.019889943
CYB5R2	-0.42914	0.000141204
DAAM2	0.292447	0.010009557
DDA1	-0.2948	0.013903692
DDX46	-0.17972	0.016536803
DECR2	-0.30405	0.014421963
DEFB7	-0.31248	0.019660939
DENND1A	0.319396	0.001222228
DERL3	0.378965	0.006317083
DEUP1	-0.13378	0.025322405
DHRS1	0.371748	0.005973553
DHX38	0.221946	0.008016623
DIP2C	0.406673	0.004739871
DNAJB6	-0.14639	0.02257771
DPYSL3	0.372341	0.010057091
DR1	-0.21653	0.005774607
DTNB	0.296432	0.004350114
DUS4L	-0.335	0.005180803
ECI1	-0.26266	0.025895152
ECM1	-0.36727	0.014313913
EDEM2	-0.2597	0.006671761
EFEMP1	-0.46693	0.000332635
EFR3B	-0.36109	0.014220842
EHMT1	0.191735	0.01674947
EIF3J	-0.18739	0.019177306
ELOVL5	-0.20735	0.007389778
ENPP1	0.328692	0.024806157
EP400	0.302758	0.018146825
EPHA2	0.325372	0.023340862
ERCC4	0.335886	0.022566723
ESPNL	0.321534	0.022920355
FABP2	0.414625	0.000329237
FAM131B	0.61906	2.55E+09
FAM151B	-0.23866	0.02386474
FAM222B	0.319831	0.015327087
FAM25A	0.383643	0.005024145
FAM96A	-0.24938	0.025927362
FAR2	-0.50147	0.000801583
FARP1	0.346538	0.02099668

FBXL3	-0.2273	0.009982608
FCER1G	-0.33235	0.026010017
FCGR2B	-0.31499	0.001308867
FCMR	0.308434	0.015556106
FDFT1	-0.31606	0.002670482
FGD1	0.300994	0.025063338
FKBP1A	-0.22515	0.02186702
FMN2	-0.24185	0.020405988
FUT8	0.219338	0.007872178
GABBR1	0.335819	0.021042146
GALNT10	0.41966	0.002061244
GALNT3	-0.28303	0.004676314
GAR1	-0.2638	0.014809243
GCLM	-0.34985	0.014339265
GLMN	-0.21129	0.026192245
GLYCTK	-0.27481	0.024013441
GNA13	0.2806	0.018925898
GNAI3	-0.25039	0.001109374
GOLPH3	-0.20055	0.02340866
GOT1L1	0.327354	0.008945742
GP5	-0.27406	0.010873376
GP9	-0.3823	0.010161895
GPR107	0.327006	0.011630036
GUCY2C	0.398338	0.007862257
HERC5	-0.34103	0.014596456
HMGN4	-0.19431	0.00320045
HR	0.36647	0.012424323
HSD17B3	0.381178	0.00891198
HSPA14	-0.1662	0.01071844
HVCN1	0.321614	0.022770057
ID3	0.397153	0.006025433
IDI1	-0.23187	0.01739073
IDO1	-0.40916	0.003262164
IFI44L	-0.29107	0.011328745
IFT122	0.379742	0.008197986
IL1A	-0.29642	0.015043544
IL1RL1	0.326701	0.023391367
IL22RA2	-0.32635	0.021428923
IL9R	0.445332	0.00179145
IRAK4	-0.34068	0.002813053
ITM2C	-0.27859	0.022365105
JAG2	0.456899	0.001016283
KCNA3	0.339597	0.021132262

KCNQ4	0.317108	0.025701572
KDELC1	0.312479	0.018736878
KLF4	-0.44322	0.002383921
KLRF1	0.418149	0.001863192
KRCC1	-0.22891	0.020134224
LACTB2	-0.26517	0.021649791
LDAH	0.301366	0.017598907
LDHA	-0.32784	0.022545726
LDLR	-0.37172	0.001700812
LGALS3	-0.19415	0.013229172
LGR6	-0.39702	0.008328534
LOC100299503; THOC2	-0.2333	0.008801053
LOC104970173; TPM1; TPM3; LOC101906472	-0.19573	0.009533991
LOC505306	0.470742	0.001685068
LOC507756	-0.7066	1.08E+07
LOC509854	0.313157	0.008944737
LOC511106	-0.21463	0.020783225
LOC515418	-0.34691	0.009634617
LOC516599	-0.2174	0.025198351
LOC534742	-0.29679	0.003294665
LOC574091	-0.35371	0.018701975
LOC617503	0.518395	6.57E+09
LOC618737	-0.45273	0.00062011
LOC786914	0.430232	0.004183325
LOC788205	0.26954	0.02277749
LOC790312	0.468579	1.65E+09
LRIG1	0.427821	0.002467585
LRP12	-0.28368	0.012178346
LRRC8A	0.270822	0.010457097
LYPD3	-0.36446	0.015224072
LYPLA1	-0.23981	0.002657179
MAP3K8	-0.36179	0.013218905
MAP4	0.208427	0.024565221
MAPK8	-0.25964	0.013142864
MAPKBP1	0.289395	0.004961749
MARC1	-0.35038	0.015012358
MARCH7	-0.25207	0.010270537
MDN1	0.304437	0.015153271
MET	0.524497	0.00039651
METAP2	-0.23097	0.006081885
METTL23	-0.2341	0.019222055
METTL4	0.340237	0.01758925

METTL9	-0.27577	0.017523952
MFAP3	-0.24923	0.019376492
MFSD14A	-0.21443	0.015117075
MFSD6L	0.366447	0.014555583
MGME1	0.235357	0.021664313
MIR222	-0.33418	0.004633546
MLLT6	0.303527	0.013840238
MLXIP	0.324352	0.002684544
MMD	-0.19989	0.011705217
MOB3B	0.3514	0.01952624
MOSPD1	-0.29088	0.009643549
MRVI1	-0.30734	0.025966483
MSMO1	-0.26965	0.012592715
MTHFD1	0.181707	0.012100442
MTOR	0.287596	0.005955446
MTUS1	-0.40281	0.001434419
MYH9	0.275806	0.007945159
MYL12B	-0.38651	0.000170018
NAA15	-0.19255	0.009049457
NAB1	-0.22003	0.026170505
NABP1	-0.24226	0.007024042
NDE1	0.365019	0.001043179
NDFIP2	-0.26556	0.011478478
NEURL1	0.299229	0.011199738
NEXN	-0.28236	0.006506785
NFKBIZ	-0.32309	0.003100614
NFRKB	0.283287	0.010407323
NHEJ1	0.315158	0.011165479
NINJ2	-0.24521	0.016453388
NKRF	-0.24218	0.021646932
NLGN3	0.358741	0.007498058
NMNAT1	-0.34085	0.011563993
NMUR2	0.296319	0.020043269
NSDHL	-0.2293	0.016672447
NUMA1	0.198594	0.004213638
NUP210	0.204711	0.012856674
OCIAD1	-0.13836	0.012363318
OSBP2	0.547389	0.000152703
OSM	-0.31193	0.023119544
OTOA	-0.39506	0.005025231
P2RY8	0.295137	0.006178585
PADI2	0.409704	0.006005974
PCYT2	-0.22789	0.007648625

PDCD6	-0.17457	0.005864562
PDE1B	0.395486	0.007230496
PDGFA	0.436056	0.003430307
PDGFRA	-0.43035	0.003292582
PDHX	-0.27525	0.010946568
PDPR	0.35683	0.003536543
PFN2	0.32306	0.023713891
PHF19	0.187583	0.019514098
PLXNB1	0.434913	0.001350529
PMS1	-0.29096	0.022280442
PNKD	-0.3432	0.011780645
PNPT1	-0.24402	0.003434513
POLR2J	-0.25668	0.008908943
POMC	-0.31968	0.019169373
PPIG	-0.2891	0.020902844
PPP1CB	-0.18179	0.013745655
PRKAR2B	0.241306	0.013052929
PRKDC	0.296073	0.008570395
PRR12	0.245804	0.023346454
PRR13	-0.29962	0.005780322
PSKH1	0.258409	0.017297859
PTGS2	-0.44737	0.002607417
PTH1R	0.379025	0.007167843
PTPRS	0.332221	0.006929404
PYCARD	-0.33469	0.021394909
RAB11FIP4	0.308773	0.009210362
RANBP9	-0.25307	8.16E+09
RBM45	-0.24715	0.014510246
RBMS2	0.291131	0.022503934
REXO5	0.383105	0.001582801
RFWD3	0.294639	0.005419586
RHBDD3	-0.22984	0.02024525
RHOB	-0.39439	0.000194325
RIC3	-0.24771	0.01081062
RIPOR3	-0.36088	0.011631721
RNF144A	0.428863	0.000720417
RNF215	0.308624	0.019952788
RNF6	-0.23875	0.023265559
RPL12	-0.31304	5.07E-107
RPL15	-0.32008	0.01128925
RPL39; LOC101902490; LOC101907518	-0.16201	0.020806129
RPTOR	0.345562	0.000140309
RREB1	0.280862	0.008093398

RSPH10B	0.370755	0.013679304
S100A1	-0.38632	0.009918093
SAMD9	-0.34469	0.021921885
SAP130	0.269189	0.011739866
SARM1	0.275337	0.020674028
SC5D	-0.27862	0.002410856
SCNM1	-0.24801	0.011533955
SCNN1A	-0.16734	0.012098931
SEPT11	0.363437	0.000980191
SERPINH1	0.368934	0.007197159
SETD1A	0.26606	0.000443593
SFI1	0.360279	0.000265868
SGPP1	-0.2612	0.001695712
SIK3	0.329959	0.002274813
SLC16A1	-0.20935	0.023442407
SLC1A4	0.378938	0.00609345
SLC33A1	-0.29894	4.14E+09
SLC39A11	0.273846	0.017411434
SLC6A4	-0.47021	0.00132992
SLC9A6	-0.21201	0.023129951
SMAD7	0.439279	0.002359315
SMC1A	0.310079	0.025834669
SMG6	0.294413	0.008606386
SMIM5	0.306625	0.018142695
SNX16	-0.34098	0.002512276
SOCS1	-0.37298	0.012910962
SOCS2	-0.39732	0.003864318
SPARC	-0.33243	0.009749803
SPATS2L	-0.3874	0.009868134
SPEG	0.447547	0.002792021
SPR	-0.33318	0.019644584
SPTLC1	-0.17907	0.020533654
SQLE	-0.2705	0.001057301
SRA1	-0.18454	0.004405047
SREBF1	-0.16536	0.023960475
SREK1IP1	-0.32881	0.020525616
SRGAP1	0.301668	0.015357788
ST3GAL1	0.303996	0.015007066
STARD3NL	-0.26254	0.010426886
SUCLA2	-0.20467	0.022126079
SYT11	-0.36011	0.01180928
SZT2	0.25612	0.021375793
TAF9B	0.300316	0.020126641

TATDN3	-0.20735	0.020848968
TBK1	-0.29656	0.014067449
TBX21	-0.33122	0.003703473
TEP1	0.266829	0.013923347
THAP2	-0.22789	0.023920423
THBD	-0.39876	0.007877151
THNSL2	0.393382	0.005982731
THY1	-0.307	0.004947411
TIAL1	-0.16714	0.019453422
TIE1	0.439181	0.003483206
TIFA	-0.3007	0.007269217
TM6SF1	-0.25848	0.008295965
TMEM144	0.388268	0.003742747
TMEM243	-0.27096	0.02024693
TMEM41B	-0.20313	0.017865244
TMEM87A	-0.1716	0.016891733
TNXB	0.334321	0.023367089
TPD52	-0.20397	0.024438924
TPP1	0.26574	0.022300486
TRA2A	-0.21772	0.00540523
TRAF3IP2	0.282855	0.018523413
TRAPPC13	-0.2282	0.015765087
TRIM35	0.227563	0.02008729
TRIM44	0.325142	0.012126106
TRMT10C	-0.2486	0.021584458
TRMT1L	-0.23701	2.27E+09
TRMT5	-0.24561	0.003653364
TRPC5	-0.25781	0.025868935
TTC1	-0.28009	4.34E+09
TTF2	0.233511	0.020114795
TUBB	0.195167	0.008370212
TXNL1	-0.21009	0.006648802
UBD	-0.30517	0.012186459
UBE2D1	-0.2303	0.014246855
UBE4B	0.182808	0.023419235
UROS	-0.2958	0.02169198
VPS37A	-0.25722	0.006013957
VPS8	0.240191	0.003719168
WBP4	-0.27434	0.001787938
WNK1	0.264065	0.012415266
WSB1	-0.23349	0.01493131
XPC	0.229898	0.005347311
XPO1	-0.18249	0.00726569

YAF2	-0.24174	0.023195826
YBEY	-0.31157	0.003534923
ZBTB33	-0.2235	0.018688699
ZBTB46	0.336758	0.021480905
ZC3H13	0.230658	0.00830117
ZC3H15	-0.35293	0.000990469
ZC3H4	0.240194	0.002587659
ZC3H7B	0.307892	0.003023118
ZFYVE26	0.23497	0.023718065
ZNF148	-0.24945	0.018718213
ZNF205	-0.37286	0.010355248
ZNF462	0.424817	0.004157365
ZNF639	-0.28727	0.005199343

Table S2. Output of statistical analyses referred to Figure 6.

	Sum of Square	df	MeanSquare	F(Dfn, Dfd)	Sig.
BetweenGroups	3.160	1	3.160	1, 16	<.0001
WithinGroups	2.360	1	2.360	1, 16	<0.001
